# Symptom-burden in people living with frailty and chronic kidney disease

**DOI:** 10.1186/s12882-020-02063-6

**Published:** 2020-09-23

**Authors:** A. C. Nixon, T. J. Wilkinson, H.M.L. Young, M. W. Taal, N. Pendleton, S. Mitra, M. E. Brady, A. P. Dhaygude, A. C. Smith

**Affiliations:** 1 Department of Renal Medicine, Lancashire Teaching Hospitals NHS Foundation Trust, Royal Preston Hospital, Sharoe Green Lane, Preston, PR2 9HT UK; 2 Centre for Health Research and Innovation, NIHR Lancashire Clinical Research Facility, Preston, UK; 3 Division of Cardiovascular Sciences, University of Manchester, Manchester, UK; 4 Department of Health Sciences, Leicester Kidney Lifestyle Team, University of Leicester, Leicester, UK; 5 NIHR Leicester Biomedical Research Centre, Leicester, UK; 6 Department of Respiratory Sciences, University of Leicester, Leicester, UK; 7 Division of Medical Sciences and Graduate Entry Medicine, University of Nottingham, Nottingham, UK; 8 Department of Renal Medicine, University Hospitals of Derby and Burton, Derby, UK; 9 Division of Neuroscience and Experimental Psychology, University of Manchester, Manchester, UK; 10 Manchester Academy of Health Sciences Centre, University of Manchester, Manchester, UK; 11 NIHR Devices For Dignity MedTech & In-vitro Diagnostics Co-operative, Manchester, UK

**Keywords:** Chronic kidney disease, Elderly, Quality of life, Frailty, Geriatric nephrology

## Abstract

**Background:**

Frailty is independently associated with worse health-related quality of life (HRQOL) in chronic kidney disease (CKD). However, the relationship between frailty and symptom experience is not well described in people living with CKD. This study’s aim was to evaluate the relationship between frailty and symptom-burden in CKD.

**Methods:**

This study is a secondary analysis of a cross-sectional observational study, the QCKD study (ISRCTN87066351), in which participants completed physical activity, cardiopulmonary fitness, symptom-burden and HRQOL questionnaires. A modified version of the Frailty Phenotype, comprising 3 self-report components, was created to assess frailty status. Multiple linear regression was performed to assess the association between symptom-burden/HRQOL and frailty. Logistic regression was performed to assess the association between experiencing symptoms frequently and frailty. Principal Component Analysis was used to assess the experienced symptom clusters.

**Results:**

A total of 353 patients with CKD were recruited with 225 (64%) participants categorised as frail. Frail participants reported more symptoms, had higher symptom scores and worse HRQOL scores. Frailty was independently associated with higher total symptom score and lower HRQOL scores. Frailty was also independently associated with higher odds of frequently experiencing 9 out of 12 reported symptoms. Finally, frail participants experienced an additional symptom cluster that included loss of appetite, tiredness, feeling cold and poor concentration.

**Conclusions:**

Frailty is independently associated with high symptom-burden and poor HRQOL in CKD. Moreover, people living with frailty and CKD have a distinctive symptom experience. Proactive interventions are needed that can effectively identify and address problematic symptoms to mitigate their impact on HRQOL.

## Background

Improved life-expectancy has been a major success of modern healthcare. However, with increased longevity comes an increased prevalence of older individuals living with multimorbidity. This trend is predicted to continue with 20.4 million people estimated to be ≥65 years old by 2066 in the UK, compared with 11.8 million in 2016 [1]. The fastest increase is predicted to be in the ≥85-year-old age group [1]. Both ageing and multimorbidity are associated with frailty, the state of vulnerability to disproportionate changes in health status when exposed to stressor events [2–4]. In Europe, the prevalence of frailty is reported to be 7.7% and pre-frailty, the precursor to frailty, 42.9% [3, 5]. Importantly for nephrology services, the prevalence of frailty appears to be far greater in those with chronic kidney disease (CKD) than in the general older population, with one study reporting a prevalence as high as 73% in dialysis dependent CKD [6].

There are two principal conceptual models of frailty, specifically the deficit accumulation model of frailty and the physical model of frailty [3, 7]. Though differing in their underlying theories, both predict vulnerability to adverse outcomes [8]. The physical model of frailty, often referred to as the Frailty Phenotype (FP), is described as a clinical syndrome involving at least three of the following components: unintentional weight loss, self-reported exhaustion, weakness (measured by grip strength), slow walking speed, and low physical activity [3]. It has a robust evidence base for predicting outcomes in patients with CKD, including increased falls, hospitalisation and mortality risk [6, 9].

People with both end-stage kidney disease and earlier stages of CKD report high symptom-burden that has a negative influence on health-related quality of life (HRQOL) [10–14]. Notably, frailty is independently associated with worse HRQOL in CKD populations [15–17]. However, the relationship between frailty and symptom experience is not well described in people living with CKD, particularly in earlier stages of CKD. Older patients and individuals with CKD prioritise outcomes relevant to daily activities and general well-being, including symptom management, over prolonged survival [18–20]. Research is needed to better understand how living with frailty and CKD influences symptomology. Targeted interventions can then be developed that are able to improve relevant patient-reported outcomes. The study’s objectives were to: (i) evaluate the relationship between frailty and symptom-burden in CKD; (ii) establish the most prevalent symptoms experienced by people with frailty and CKD; and (iii) assess the symptom-clusters experienced by non-frail and frail people with CKD.

## Methods

### Study design, setting and participant selection

This study is a secondary analysis of the QCKD study data (ISRCTN87066351), which was a cross-sectional observational study that aimed to improve understanding of physical activity behaviour across CKD stages [21]. Data presented in this analysis were gathered between February 2018 and October 2018. Ethical approval was granted by the East Midlands-Leicester South Research Ethics Committee and Health Research Authority (reference: 12/EM/0184). Written informed consent was obtained from all participants and the study was conducted in compliance with the Declaration of Helsinki. Potential participants were identified from a general practitioner (GP) practice in the Leicester region, United Kingdom (UK), and from the Renal Risk in Derby (RRID) study population [22], which originally recruited participants from 32 primary care clinics in Derbyshire, UK. Participants aged ≥18 years old with two estimated glomerular filtration rate (eGFR) values < 60 ml/min/1.73m^2^ more than 90 days apart and able to give informed consent were eligible for inclusion.

### Outcome measures

Participants were asked to complete a survey pack that contained study outcome measures. Recruitment was via two routes: in the GP practice, eligible patients were identified by practice staff, and participants in the RRID study, who had previously been recruited from GP practices [22], were screened by the Chief Investigator and research staff. In both cases, potential participants were sent study packs containing an invitation letter and information sheet, consent form and the survey pack for completion, together with a post-paid return envelope. The demographic and clinical characteristics section of the survey pack asked participants to report their age, sex, smoking history and highest level of educational attainment. Participants were also asked to report the presence of health problems. Haemoglobin, eGFR and albumin laboratory variables were obtained from medical records with informed consent. The survey pack also contained the following questionnaires: General Practice Physical Activity Questionnaire (GPPAQ), Duke Activity Status Index (DASI), Short Form-12 (SF-12) and Kidney Symptom Questionnaire (KSQ).

The GPPAQ is a questionnaire developed by the Department of Health to assess levels of physical activity [23, 24]. The GPPAQ consists of questions about both work and domestic physical activities and perceived walking pace. Responses are used to calculate a 4-level physical activity index reflecting current physical activity. Participants are categorised as either ‘active’, ‘moderately active’, ‘moderately inactive’ or ‘inactive’.

The DASI is a questionnaire that measures functional capacity [25, 26]. It comprises 12-items that assess perceived ability to perform activities of daily living. Responses are used to calculate a raw DASI score, with higher scores indicating greater functional capacity. As previously validated [26], scores were transformed into estimated peak oxygen uptake (VO_2_ peak) values to provide a measure of cardiopulmonary fitness.

The SF-12 encompasses 12 questions that are used to assess HRQOL [11, 27]. Responses can be used to construct an 8-scale profile of health and well-being and to generate physical and mental health summary measures: Physical Component Summary (PCS) and Mental Component Summary (MCS). Higher scores represent better HRQOL.

The KSQ is a questionnaire that measures participant symptom perception [10, 28, 29]. The updated version was used to assess the frequency of 13 symptoms on a five-point scale: ‘never’ (0), ‘less than once a week’ (1), ‘1-2 times a week’ (2), ‘several times a week’ (3), or ‘every day’ (4). The total frequency score was used to assess total symptom-burden with higher scores denoting greater symptom-burden. In this study, individual symptoms were defined as experienced frequently if participants reported experiencing the symptom ‘several times a week’ or ‘every day’.

### Frailty

Woods et al. demonstrated that a modified version of the FP, which substituted measures of grip strength and walking speed for self-report, independently predicted adverse outcomes, including hospitalisation, disability and mortality [30]. Studies have since shown that modified versions of the FP also predict adverse outcomes in CKD cohorts [31–34]. Delgado et al. demonstrated that a modified FP was associated with mortality in participants with non-dialysis dependent CKD [33]. Johansen et al. determined that a modified FP containing three-components (specifically weakness/slowness, low physical activity and “undernourished” with each assigned 1 point) was also associated with mortality in patients receiving dialysis [31]. Our previous work revealed that the weight loss FP component is not a significant contributor to HRQOL in patients with CKD, whereas the exhaustion component is associated with worse scores across all domains of HRQOL [15]. Bao et al. used a three-component modified FP that included slowness/weakness, low physical activity and exhaustion components and considered participants with two or more components as frail [32]. This modified FP was also associated with mortality in patients receiving dialysis [32]. Comparable to these previous studies, we used a modified version of the FP comprising three self-report components to assess frailty status: 1) weakness/slowness defined as a SF-12 Physical Functioning score < 75; 2) low physical activity as ‘inactive’ by the GPPAQ; and 3) exhaustion as a SF-12 Vitality score < 55. Each component was assigned 1 point and a participant was categorised as frail if two or more components were present.

### Statistical methods

As a secondary analysis, no a priori sample size calculation was performed. All statistical analyses were performed using IBM SPSS version 25 (IBM, USA). Multiple imputation was performed for data assumed to be either missing completely at random or missing at random [35]. Missing ethnicity data and KSQ libido item scores were assumed to be missing not at random and therefore were not imputed. As recommended by Graham et al. [36], the number of imputations (20 imputations) performed was based on the fraction of missing information.

Descriptive statistics were used to describe demographic and clinical characteristics. Pooled mean and standard error (SE) are reported for imputed continuous variables. Categorical variables are presented as frequencies and percentages. Differences in continuous variables for participants categorised as non-frail and frail were assessed using the Independent t test. Multiple linear regression was used to assess the association between symptom-burden (KSQ total frequency score) and HRQOL (SF-12 PCS and MCS scores) and frailty status (frail vs. non-frail), demographics (age and sex) and clinical parameters (eGFR and haemoglobin). Binomial logistic regression was used to assess the association between experiencing symptoms frequently and frailty status (frail vs. non-frail), demographics (age and sex) and clinical parameters (eGFR and haemoglobin). Independent variables included in the regression analyses were selected a priori. To minimise the risk of a type I error, a Bonferroni correction was applied to an alpha level of 0.05 to determine the level of significance for each statistical test.

Principal Component Analysis (PCA) was run on the KSQ symptom frequency scores to assess the symptom clusters experienced by participants categorised as non-frail and frail. PCA was performed on complete cases only. Only variables with at least one correlation coefficient ≥ 0.3 in the correlation matrix were included in the analysis. A minimum overall Kaiser-Meyer-Olkin (KMO) value of 0.6 and a minimum individual KMO value of 0.5 were used to determine sampling adequacy. Bartlett’s test of sphericity was used to indicate that data was suitable for PCA. An eigenvalue-one criterion was used to determined how many components to retain. Furthermore, Varimax rotation was used and symptoms that loaded on more than one component (using a minimum coefficient cut-off of 0.5) were removed from the analysis to create a ‘simple structure’ and aid interpretability.

## Results

### Participant characteristics

**Fig. 1:**
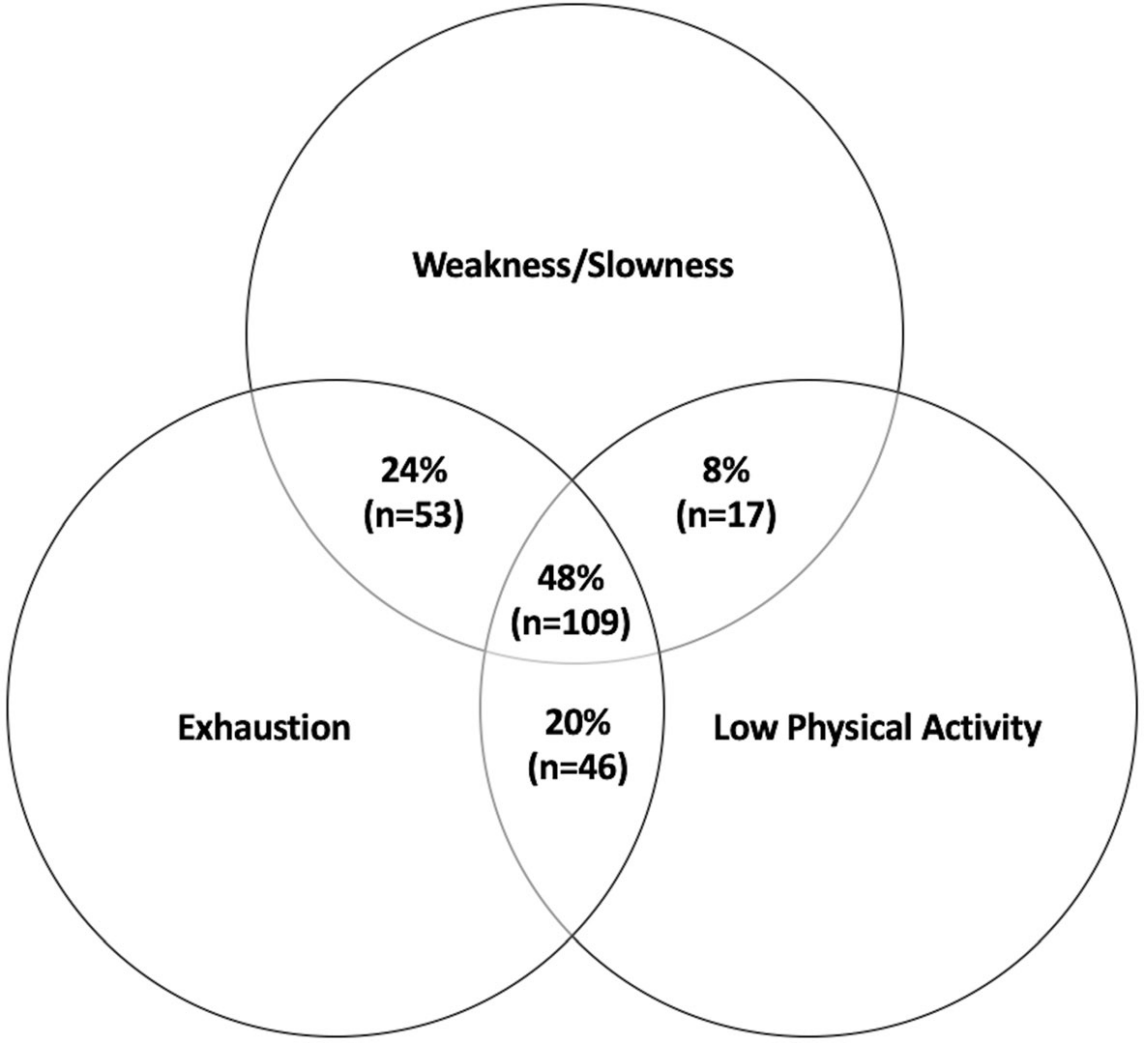
Overlap of Modified Frailty Phenotype Components for Participants Categorised as Frail

Complete data were available to evaluate the frailty status of 255 participants out of a total of 353 participants. Missing data frequency is presented in the Supplementary Materials File (Table S1). An analysis of complete cases is also presented in the Supplementary Materials File (Tables S2, S3, S4 and S5). Following multiple imputation, frailty status was calculated for all 353 participants. Two hundred and twenty-five (64%) participants were categorised as frail and 128 (36%) participants categorised as non-frail. Participant demographics and clinical characteristics are reported in Table 1. Frail participants were significantly older and had a lower eGFR, albumin concentration and estimated VO_2_ peak than non-frail participants. Furthermore, frail participants reported more health problems than non-frail participants. Figure 1 demonstrates the overlap of the modified FP components for participants categorised as frail.

**Table 1:** Participant Baseline Demographic and Clinical Characteristics

	Non-Frail (*n* = 128)	Frail (*n* = 225)	Unadjusted *P* Value
Age (years), mean (SE)	71.5 (0.9)	77.7 (0.6)	< 0.001^*^
Female, n (%)	70 (55)	130 (58)	
Current or ex-smoker, n (%)	57 (45)	125 (56)	
University/college qualification, n (%)	43 (34)	57 (25)	
Self-reported health problems, n (%)			
- Diabetes	13 (10)	45 (20)	
- Heart	19 (15)	84 (37)	
- Stroke	6 (5)	32 (14)	
- Blood vessels or circulation	19 (15)	66 (29)	
- Lung or breathing	17 (13)	64 (28)	
- Liver	7 (5)	16 (7)	
- Joints, bones or muscles	52 (41)	161 (72)	
- Mental health	14 (11)	37 (16)	
Chronic kidney disease stage, n (%)			
- CKD G2	14 (11)	17 (8)	
- CKD G3a	87 (68)	123 (55)	
- CKD G3b	26 (20)	68 (30)	
- CKD G4	1 (1)	17 (8)	
Laboratory Variables, mean (SE)			
- eGFR (mL/min/1.73m^2^)	50.9 (0.7)	45.8 (0.7)	< 0.001^*^
- Haemoglobin (g/L)	137.4 (1.7)	132.2 (1.1)	0.01
- Albumin (g/L)	41.4 (0.5)	39.2 (0.4)	< 0.001^*^
Frailty Components, n (%)			
- Weakness/slowness	12 (9)	179 (80)	
- Exhaustion	41 (32)	208 (92)	
- Low physical activity	32 (25)	172 (76)	
Slow walking pace, n (%)	11 (9)	130 (58)	
VO_2_ Peak mL/kg/min, mean (SE)	33.9 (6.0)	21.7 (9.1)	< 0.001^*^
Total symptoms, mean (SE)	6.9 (0.3)	9.2 (0.1)	< 0.001^*^
Total symptom score, mean (SE)	14.6 (0.7)	24.9 (0.6)	< 0.001^*^
SF-12 PCS, mean (SE)	51.8 (0.6)	36.4 (0.7)	< 0.001^*^
SF-12 MCS, mean (SE)	53.8 (0.7)	50.2 (0.7)	< 0.001^*^

Data presented as mean (SE) or frequencies (%). ^*^Remain statistically significant following Bonferroni correction. Estimated VO_2_ Peak, (peak oxygen uptake). SF-12, Short Form-12

### Symptom-burden and HRQOL in participants with frailty and CKD

Frail participants reported more symptoms and had a significantly higher KSQ total symptom score than non-frail participants (Table 1). In addition, frail participants had significantly lower SF-12 PCS and MCS scores. Frailty, when adjusted for age, sex, eGFR and haemoglobin, was associated with higher total symptom score and lower PCS and MCS scores (Table 2). Moreover, frailty was associated with a 9-point higher total symptom score. Lower eGFR was associated with higher total symptom score and lower PCS score. Older age was associated with lower PCS score. Female participants had higher total symptom scores and lower MCS score.

**Table 2:** Association Between Frailty, Symptom-Burden and Health-Related Quality of Life

	Unstandardised β Coefficient	SE	Unadjusted *P* Value
KSQ total frequency score			
- Frailty	9.28	1.01	< 0.001^*^
- Age	0.04	0.05	0.39
- Female	3.69	1.00	< 0.001^*^
- eGFR	−0.15	0.05	0.004^*^
Haemoglobin	0.03	0.03	0.41
SF-12 PCS score			
- Frailty	−13.57	1.12	< 0.001^*^
- Age	−0.18	0.06	0.002^*^
- Female	−2.19	1.11	0.05
- eGFR	0.16	0.06	0.007^*^
- Haemoglobin	−0.02	0.03	0.48
SF-12 MCS score			
- Frailty	−3.85	1.14	0.001^*^
- Age	0.13	0.06	0.03
- Female	−3.80	1.09	< 0.001^*^
- eGFR	0.07	0.06	0.23
- Haemoglobin	0.01	0.03	0.80

^*^Remain statistically significant following Bonferroni correction

*KSQ* Kidney Symptom Questionnaire, *SF-12* Short Form-12

### Symptoms experienced frequently by participants with frailty and CKD

Frail participants reported experiencing all symptoms more frequently than non-frail participants (Table 3). The three most often reported symptoms frequently experienced for both frail and non-frail participants included bone/joint pain (frail 69%; non-frail 34%), urinary frequency (frail 64%; non-frail 50%) and tiredness (frail 61%; non-frail 31%). The greatest differences between frail and non-frail participants were seen with loss of muscle strength, bone/joint pain, breathlessness, tiredness and cramp/muscle stiffness. Frailty, when adjusted for age, sex, eGFR and haemoglobin, was associated with higher odds of frequently experiencing all symptoms, except loss of appetite, restless legs and urinary frequency (Table 4). After applying a Bonferroni correction, lower eGFR and older age were not associated with higher odds of frequently experiencing any specific symptom. Female sex was associated with higher odds of frequently experiencing sleep disturbance and bone/joint pain.

**Table 3:** Symptoms Experienced Frequently by Non-Frail and Frail Participants

	Non-Frail (*n* = 128)	Frail (*n* = 225)	Difference in Frail vs. Non-Frail (%)
Loss of muscle strength, n (%)	20 (16)	125 (56)	↑40%
Bone/joint pain, n (%)	44 (34)	155 (69)	↑35%
Breathlessness, n (%)	13 (10)	94 (42)	↑32%
Tiredness, n (%)	40 (31)	137 (61)	↑30%
Cramp/muscle stiffness, n (%)	20 (16)	98 (44)	↑28%
Itching, n (%)	22 (17)	92 (41)	↑24%
Sleep disturbance, n (%)	35 (27)	113 (50)	↑23%
Feeling cold, n (%)	32 (25)	109 (48)	↑23%
Poor concentration, n (%)	11 (9)	61 (27)	↑18%
Urinary frequency, n (%)	64 (50)	145 (64)	↑14%
Restless legs, n (%)	18 (14)	59 (26)	↑12%
Loss of appetite, n (%)	3 (2)	29 (13)	↑11%

Data presented as frequencies (%)

**Table 4:** Association Between Frailty and Symptoms Frequently Experienced

	SE	Unadjusted *P* Value	Odds Ratio	95% CI
Itching				
- Frailty	0.31	< 0.001^*^	3.17	1.72–5.84
- Age	0.02	0.89	1.00	0.97–1.03
- Female	0.28	0.04	0.56	0.32–0.97
- eGFR	0.01	0.48	0.99	0.97–1.02
- Haemoglobin	0.01	0.37	0.99	0.98–1.01
Sleep disturbance				
- Frailty	0.29	< 0.001^*^	2.97	1.68–5.25
- Age	0.01	0.61	0.99	0.97–1.02
- Female	0.27	0.002^*^	2.34	1.38–3.96
- eGFR	0.01	0.37	0.99	0.96–1.02
- Haemoglobin	0.01	0.05	1.02	1.00–1.04
Loss of appetite				
- Frailty	0.79	0.07	4.34	0.91–20.70
- Age	0.03	0.34	1.03	0.97–1.08
- Female	0.48	0.14	2.04	0.80–5.17
- eGFR	0.02	0.05	0.96	0.92–1.00
- Haemoglobin	0.01	0.17	0.98	0.96–1.01
Tiredness				
- Frailty	0.28	< 0.001^*^	3.90	2.25–6.78
- Age	0.01	0.02	0.97	0.94–0.99
- Female	0.26	0.01	1.97	1.17–3.30
- eGFR	0.01	0.07	0.98	0.95–1.00
- Haemoglobin	0.01	0.81	1.00	0.98–1.01
Bone/joint pain				
- Frailty	0.26	< 0.001^*^	3.56	2.14–5.92
- Age	0.01	0.29	1.02	0.99–1.04
- Female	0.26	0.007^*^	2.03	1.21–3.40
- eGFR	0.01	0.06	0.98	0.95–1.00
- Haemoglobin	0.01	0.72	1.00	0.98–1.01
Poor concentration				
- Frailty	0.41	0.001^*^	3.94	1.75–8.87
- Age	0.02	0.66	1.01	0.97–1.04
- Female	0.33	0.06	1.87	0.98–3.56
- eGFR	0.02	0.48	0.99	0.96–1.02
- Haemoglobin	0.01	0.25	1.01	0.99–1.04
Loss of muscle strength				
- Frailty	0.32	< 0.001^*^	4.94	2.61–9.35
- Age	0.02	0.02	1.04	1.01–1.07
- Female	0.29	0.23	1.42	0.80–2.51
- eGFR	0.02	0.04	0.97	0.94–1.00
- Haemoglobin	0.01	0.57	1.01	0.99–1.02
Breathlessness				
- Frailty	0.40	< 0.001^*^	5.65	2.56–12.48
- Age	0.02	0.29	1.02	0.98–1.05
- Female	0.29	0.27	1.38	0.78–2.43
- eGFR	0.01	0.68	0.99	0.97–1.02
- Haemoglobin	0.01	0.06	0.99	0.97–1.00
Cramp/muscle stiffness				
- Frailty	0.34	< 0.001^*^	4.25	2.19–8.24
- Age	0.02	0.86	1.00	0.97–1.03
- Female	0.29	0.03	1.90	1.07–3.37
- eGFR	0.02	0.26	0.98	0.96–1.01
- Haemoglobin	0.01	0.08	1.02	1.00–1.04
Restless legs				
- Frailty	0.35	0.03	2.10	1.06–4.17
- Age	0.02	0.59	1.01	0.98–1.04
- Female	0.31	0.91	1.04	0.57–1.89
- eGFR	0.02	0.78	1.00	0.97–1.04
- Haemoglobin	0.01	0.86	1.00	0.98–1.02
Feeling cold				
- Frailty	0.29	0.002^*^	2.45	1.39–4.31
- Age	0.01	0.59	1.01	0.98–1.03
- Female	0.25	0.15	1.45	0.88–2.38
- eGFR	0.01	0.20	0.98	0.96–1.01
- Haemoglobin	0.01	0.91	1.00	0.98–1.02
Urinary frequency				
- Frailty	0.29	0.13	1.54	0.88–2.71
- Age	0.01	0.05	1.03	1.00–1.06
- Female	0.26	0.58	1.15	0.70–1.91
- eGFR	0.01	0.44	0.99	0.96–1.02
- Haemoglobin	0.01	0.30	1.01	0.99–1.02

^*^Remain statistically significant following Bonferroni correction

### Symptom clusters in non-frail and frail participants with CKD

The suitability of PCA was assessed prior to analysis as outlined in the statistical methods section. The overall KMO for frail and non-frail groups was 0.78 and 0.64, respectively. The minimum individual KMO for frail and non-frail groups was 0.69 and 0.59, respectively. Table 5 illustrates the 2 symptom clusters experienced by non-frail participants and the 3 symptom clusters experienced by frail participants. There were symptom clusters associated with sleep disturbance and musculoskeletal symptoms for both non-frail and frail participants. Frail participants experienced an additional symptom cluster that included the following symptoms: loss of appetite, tiredness, feeling cold and poor concentration.

**Table 5:** Principal Component Analysis of Non-Frail and Frail Participant Symptom Scores

**A. Non-Frail**				
Cluster	Symptoms	Rotated Component Coefficient		
		**1**	**2**	
Sleep disturbance	Sleep disturbance	0.874		
	Restless legs	0.783		
	Poor concentration	0.645		
Musculoskeletal/breathlessness	Breathlessness		0.778	
	Bone/joint pain		0.764	
	Cramp/muscle stiffness		0.687	
**B. Frail**				
Cluster	Symptoms	Rotated Component Coefficient		
		**1**	**2**	**3**
Frailty	Loss of appetite	0.715		
	Tiredness	0.697		
	Feeling cold	0.566		
	Poor concentration	0.503		
Sleep disturbance	Urinary frequency		0.747	
	Sleep disturbance		0.665	
	Restless legs		0.647	
	Breathlessness		0.503	
Musculoskeletal	Cramp/muscle stiffness			0.865
	Loss of muscle strength			0.627
	Bone/joint pain			0.551

## Discussion

To our knowledge, this is the first study to explore symptom experience and its relationship to frailty status in patients with CKD. High symptom burden is described in patients with CKD, particularly pre-dialysis and dialysis dependent CKD [14]. Our study demonstrates that patients with earlier CKD stages also have high symptom-burden. Both renal function and frailty status are independently associated with symptom-burden. However, frailty was associated with a 9.3-point point higher total symptom score, whereas a decrease of 10 ml/min/1.73m^2^ in eGFR was only associated with a 1.5-point higher total symptom score. Given the high symptom-burden reported in participants living with frailty and CKD, the associated worse HRQOL, particularly physical HRQOL, is unsurprising. Perhaps reflecting an associated decline in functional ability, older age was associated with a lower SF-12 PCS score. As reported in previous studies, female sex was independently associated with worse HRQOL [37, 38], specifically lower SF-12 MCS scores, which corresponds with the greater symptom-burden reported by female participants.

Frailty was independently associated with two- to over five-fold higher odds of experiencing all symptoms frequently, except loss of appetite, restless legs and urinary frequency. There was over four-fold higher odds of frequently experiencing breathlessness, loss of muscle strength and cramps/muscle stiffness for participants categorised as frail. Both non-frail and frail participants had a symptom-cluster containing symptoms that may contribute to or be the result of sleep disturbance, including restless legs, urinary frequency, poor concentration and sleep disturbance itself. Furthermore, both non-frail and frail participants had a symptom-cluster containing musculoskeletal symptoms including cramp/muscle stiffness and bone/joint pain. These symptoms are often reported in people with CKD, whether receiving dialysis or not [10, 14]. However, the clusters described in our study are different to other reports in CKD populations [39–42], which may reflect differences in participant demographics and characteristics. We report symptom-clusters in earlier stages of CKD with a mean eGFR of 51 and 46 ml/min/1.73m^2^ in non-frail and frail participants, respectively. Symptom-clusters may evolve as kidney function declines and symptom-burden increases. Further research is needed on the stability of symptom-clusters with worsening kidney function.

To our knowledge, no studies have previously explored symptom-clusters in patients living with frailty with or without CKD. Within our study, participants categorised as frail experienced an additional symptom-cluster comprising loss of appetite, tiredness, feeling cold and poor concentration. These symptoms may be a consequence of the frailty syndrome itself and were therefore termed the ‘frailty’ symptom-cluster. Weight loss is a component of the original FP and likely the result of chronic under-nutrition and progressive sarcopenia [2, 3, 9]. Self-reported exhaustion is also a component of the FP [3]. Our previous work demonstrated that it is the most important FP component contributing to HRQOL in patients living with frailty and CKD [15]. In this latest study, frail participants had a lower estimated VO_2_ peak than non-frail participants, which may be linked to symptoms of exhaustion or tiredness [3]. The symptom-cluster analysis findings highlight that symptom experience may not be uniform in CKD populations. Holistic assessment, particularly for individuals identified as frail, may aid the identification of problematic symptoms.

The Comprehensive Geriatric Assessment (CGA) is the gold standard of care of the older patient living with frailty [43]. It is ‘a multidimensional, multidisciplinary process which identifies medical, social and functional needs, and the development of an integrated/co-ordinated care plan to meet those needs’ [43]. Frailty is increasingly recognised in specialised medicine, including nephrology, and the model of care for patients living with frailty and CKD is evolving [8, 44]. The CGA, or modified versions, have been successfully used in CKD populations to identify geriatric impairments and associated symptoms [44–46].

Most participants within our study had CKD G3, patients with this degree of renal impairment are usually managed by healthcare professionals other than nephrologists, often primary care practitioners. Further research is needed into the most effective approach to implementing the CGA in different care settings, including primary care [47]. However, this holistic person-centred approach to care may lead to improved symptom management and better HRQOL in patients living with frailty and CKD.

There are acknowledged limitations to this study. The Physical Functioning and Vitality SF-12 scores were used within the composite modified FP assessment and within generation of the SF-12 PCS and MCS scores. This overlap may affect the interpretability of the association between the frailty and HRQOL scores. Furthermore, our modified FP measure dichotomised participants into non-frail and frail groups and did not identify pre-frail individuals who may have been incorporated within the non-frail group. Frailty measures that have finer granularity, such as the Frailty Index, can more precisely define risk for any given individual [48]. Regardless, our modified FP was comparable to that used by other authors that have demonstrated an association with outcomes [31–34]. We report a higher prevalence of frailty than previous studies in non-dialysis CKD populations [6]. Self-report frailty assessment methods are at risk of over-estimating frailty status [49]. Nevertheless, our modified FP was independently associated high symptom-burden. Furthermore, participants categorised as frail were older, reported a higher number of health problems, had lower eGFR, lower albumin concentration and lower functional ability, all of which are reported in patients living with frailty [3, 6, 7, 50]. Thus, we consider our modified FP a pragmatic method to identify patients at risk of frailty and high symptom-burden. This study’s cross-sectional design does not allow conclusions to be made on longitudinal changes in and underlying causation of symptom experience. It also relied on self-report and willingness to complete and return a survey pack. There were associated risks of incomplete survey return, question misinterpretation, recall bias and sampling bias. Finally, this study involved an older population with early stage CKD and did not describe ethnicity, therefore results may not be generalizable to other populations, including those with more advanced CKD.

## Conclusions

In conclusion, this secondary analysis has highlighted that frailty is independently associated with high symptom-burden and poor HRQOL in people with CKD. Moreover, people living with frailty and CKD have a distinctive symptom experience. Proactive interventions are needed that can effectively identify and address problematic symptoms to mitigate their impact on HRQOL. We suggest future research evaluates holistic person-centred models of care for people living with frailty and CKD.

## Supplementary information

**Additional file 1: Table S1.** Missing Data Frequencies. **Table S2.** Complete Cases: Participant Baseline Demographic and Clinical Characteristics. **Table S3.** Complete Cases: Symptoms Experienced Frequently by Non-Frail and Frail Participants. **Table S4.** Complete Cases: Association Between Frailty, Symptom-Burden and Health-Related Quality of Life. **Table S5.** Complete Cases: Association Between Frailty and Symptoms Frequently Experienced.

## Data Availability

The datasets used and/or analysed during the current study are available from the corresponding author on reasonable request.

## Abbreviations

CKD: Chronic Kidney Disease
CGA: Comprehensive Geriatric Assessment
DASI: Duke Activity Status Index
eGFR: Estimated Glomerular Filtration Rate
FP: Frailty Phenotype
GPPAQ: General Practice Activity Questionnaire
HRQOL: Health-Related Quality of Life
KMO: Kaiser-Meyer-Olkin
LTHTR: Lancashire Teaching Hospitals NHS Foundation Trust
KSQ: Kidney Symptom Questionnaire
MCS: Mental Component Summary
NHS: National Health Service
NICE: National Institute for Health and Care Excellence
NIHR: National Institute of Health Research
PCA: Principal Component Analysis
PCS: Physical Component Summary
SE: Standard Error
SF-12: Short Form-12v2
VO_2_ Peak: Peak oxygen uptake
