# Supplementary material for: Symptom-burden in people living with frailty and chronic kidney disease
Source: BMC Nephrol. 2020 Sep 23;21:411. doi: 10.1186/s12882-020-02063-6 (PMC7513484; doi:10.1186/s12882-020-02063-6)
Supplement: Supplementary file 1 — Additional file 1: Table S1. Missing Data Frequencies. Table S2. Complete Cases: Participant Baseline Demographic and Clinical Characteristics. Table S3. Complete Cases: Symptoms Experienced Frequently by Non-Frail and Frail Participants. Table S4. Complete Cases: Association Between Frailty, Symptom-Burden and Health-Related Quality of Life. Table S5. Complete Cases: Association Between Frailty and Symptoms Frequently Experienced. [file 12882_2020_2063_MOESM1_ESM.docx]

**Supplementary MATERIALS**

**Supplementary Table 1. Missing Data Frequencies.**

|  | Frequency | Percent |
| --- | --- | --- |
| Ethnicity | 288 | 81.6 |
| KSQ libido | 105 | 29.7 |
| GPPAQ physical activity index | 84 | 23.8 |
| KSQ urinary frequency | 56 | 15.9 |
| KSQ loss of muscle strength | 53 | 15.0 |
| KSQ loss of appetite | 48 | 13.6 |
| KSQ restless legs | 43 | 12.2 |
| KSQ poor concentration | 43 | 12.2 |
| KSQ itching | 41 | 11.6 |
| KSQ cramp/muscle stiffness | 40 | 11.3 |
| KSQ breathlessness | 39 | 11.0 |
| KSQ feeling cold | 38 | 10.8 |
| KSQ sleep disturbance | 33 | 9.3 |
| Albumin | 27 | 7.6 |
| KSQ tiredness | 24 | 6.8 |
| KSQ bone/joint pain | 22 | 6.2 |
| Haemoglobin | 22 | 6.2 |
| SF-12 MCS score | 21 | 5.9 |
| SF-12 PCS score | 21 | 5.9 |
| SF-12 vitality score | 21 | 5.9 |
| Self-reported slow walking pace | 15 | 4.2 |
| eGFR | 15 | 4.2 |
| Age | 14 | 4.0 |
| Self-reported lung or breathing problem | 13 | 3.7 |
| SF-12 physical functioning score | 11 | 3.1 |
| Self-reported mental health problem | 11 | 3.1 |
| Self-reported Joint, bone or muscle problem | 11 | 3.1 |
| Self-reported blood vessel or circulatory problem | 11 | 3.1 |
| Self-reported heart problem | 11 | 3.1 |
| Self-reported Liver problem | 10 | 2.8 |
| Self-reported stroke | 10 | 2.8 |
| Educational qualification | 9 | 2.5 |
| Self-reported diabetes | 9 | 2.5 |
| Smoking history | 6 | 1.7 |
| Sex | 5 | 1.4 |
| DASI Score | 3 | 0.8 |

GPPAQ, General Practice Activity Questionnaire. KSQ, Kidney Symptom Questionnaire. SF-12, Short Form-12. MCS, Mental Component Summary. PCS, Physical Component Summary. eGFR, estimated Glomerular Filtration Rate. DASI, Duke Activity Status Index.

**Supplementary Table 2. Complete Cases: Participant Baseline Demographic and Clinical Characteristics.**

|  | Non-Frail | Frail | Unadjusted  P Value |
| --- | --- | --- | --- |
| Age (years), mean (SD), NF n=72, F n=178 | 70.0 (10.4) | 77.7 (8.9) | <0.001 |
| Female, n (%), NF n=74, F n=180 | 40 (54) | 100 (56) | 0.83 |
| Current or ex-smoker, n (%), NF n=74, F n=180 | 30 (41) | 101 (56) | 0.02 |
| University/college qualification, n (%), NF n=74, F n=176 | 27 (36) | 46 (26) | 0.10 |
| Self-reported health problems, n (%)   - Diabetes, NF n=74, F n=178 - Heart, NF n=74, F n=178 - Stroke, NF n=74, F n=178 - Blood vessels or circulation, NF n=74, F n=177 - Lung or breathing, NF n=73, F n=178 - Liver, NF n=74, F n=178 - Joints, bones or muscles, NF n=74, F n=177 - Mental health, NF n=74, F n=177 | 8 (11)  9 (12)  3 (4)  13 (18)  11 (15)  6 (8)  31 (42)  13 (14) | 33 (19)  61 (34)  21 (12)  53 (30)  51 (29)  10 (6)  128 (72)  27 (15) | 0.13  <0.001  0.06  0.04  0.02  0.57  <0.001  0.72 |
| Chronic kidney disease stage, n (%), NF n=74, F n=170   - CKD G2 - CKD G3a - CKD G3b - CKD G4 | 8 (11)  50 (68)  15 (20)  1 (1) | 15 (9)  87 (51)  53 (31)  15 (9) | 0.63  0.02  0.08  0.045 |
| Laboratory Variables, mean (SD)   - eGFR (mL/min/1.73m^2^), NF n=74, F n=170 - Haemoglobin (g/L), NF n=72, F n=167 - Albumin (g/L), NF n=70, F n=165 | 50.5 (8.4)  137.0 (21.1)  42.2 (4.8) | 45.5 (11.3)  131.0 (16.5)  39.1 (5.5) | <0.001  0.02  <0.001 |
| Frailty Components, n (%), NF n=75, F n=180   - Weakness/slowness - Exhaustion - Low physical activity | 3 (4)  22 (29)  29 (39) | 136 (76)  164 (91)  164 (91) | <0.001  <0.001  <0.001 |
| Slow walking pace, n (%), NF n=75, F n=179 | 3 (4) | 99 (55) | <0.001 |
| Estimated VO_2_ Peak, mean (SD), NF n=75, F n=180 | 33.9 (6.0) | 21.7 (9.1) | <0.001 |
| Total symptoms, mean (SD), NF n=59, F n=118 | 6.2 (2.8) | 9.1 (2.2) | <0.001 |
| Total symptom score, mean (SD), NF n=59, F n=118 | 12.7 (7.3) | 23.9 (9.1) | <0.001 |
| SF-12 PCS, mean (SD), NF n=75, F n=179 | 52.6 (5.6) | 37.4 (10.9) | <0.001 |
| SF-12 MCS, mean (SD), NF n=75, F n=179 | 53.9 (6.0) | 50.1 (10.0) | <0.001 |

Data presented as mean (SD) or frequencies (%). NF, Non-Frail. F, Frail. VO_2_ Peak, (peak oxygen uptake). SF-12, Short Form-12.

**Supplementary Table 3. Complete Cases: Symptoms Experienced Frequently by Non-Frail and Frail Participants.**

|  | Non-Frail | Frail | Unadjusted P Value | Difference in Frail vs. Non-Frail (%) |
| --- | --- | --- | --- | --- |
| Loss of muscle strength, n (%), NF n=70, F n= 158 | 8 (11) | 81 (51) | <0.001 | ↑40 |
| Bone/joint pain, n (%), NF n=74, F n=174 | 24 (32) | 117 (67) | <0.001 | ↑35 |
| Breathlessness, n (%), NF n=70, F n=166 | 6 (9) | 66 (40) | <0.001 | ↑31 |
| Feeling cold, n (%), NF n=70, F n=169 | 11 (16) | 78 (46) | <0.001 | ↑30 |
| Tiredness, n (%), NF n=72, F n=172 | 23 (32) | 103 (60) | <0.001 | ↑28 |
| Cramp/muscle stiffness, n (%), NF n=69, F n=167 | 10 (14) | 67 (40) | <0.001 | ↑26 |
| Sleep disturbance, n (%), NF n=72, F n=169 | 19 (26) | 81 (48) | 0.002 | ↑22 |
| Itching, n (%), NF n=71, F n=165 | 13 (18) | 63 (38) | 0.003 | ↑20 |
| Poor concentration, n (%), NF n=71, F n=163 | 4 (6) | 38 (23) | 0.001 | ↑17 |
| Urinary frequency, n (%), NF n=66, F n=156 | 30 (45) | 94 (60) | 0.04 | ↑15 |
| Loss of appetite, n (%), NF n= 72, F n=159 | 2 (3) | 19 (12) | 0.03 | ↑9 |
| Restless legs, n (%), NF n=72, F n=162 | 12 (17) | 37 (23) | 0.28 | ↑6 |

Data presented as frequencies (%). NF, Non-Frail. F, Frail.

**Supplementary Table 4. Complete Cases: Association Between Frailty, Symptom-Burden and Health-Related Quality of Life.**

|  | Unstandardised  β Coefficient | SE | Standardised  β Coefficient | Unadjusted  P Value |
| --- | --- | --- | --- | --- |
| KSQ total frequency score   - Frailty - Age - Female - eGFR - Haemoglobin | 10.40  -0.05  5.58  -0.24  0.08 | 1.48  0.07  1.40  0.06  0.04 | 0.49  -0.05  0.28  -0.26  0.14 | <0.001  0.52  <0.001  <0.001  0.05 |
| SF-12 PCS score   - Frailty - Age - Female - eGFR - Haemoglobin | -13.61  -0.12  -2.29  0.21  -0.04 | 1.49  0.07  1.36  0.06  0.04 | -0.52  -0.10  -0.09  0.19  -0.07 | <0.001  0.08  0.09  0.001  0.25 |
| SF-12 MCS score   - Frailty - Age - Female - eGFR - Haemoglobin | -5.24  0.19  -3.29  0.07  0.01 | 1.40  0.07  1.27  0.06  0.04 | -0.26  0.21  -0.18  0.08  0.02 | <0.001  0.005  0.01  0.27  0.81 |

KSQ, Kidney Symptom Questionnaire. SF-12, Short Form-12.

**Supplementary Table 5. Complete Cases: Association Between Frailty and Symptoms Frequently Experienced.**

|  | SE | Unadjusted  P Value | Odds Ratio | 95% CI |
| --- | --- | --- | --- | --- |
| Itching   - Frailty - Age - Female - eGFR - Haemoglobin | 0.41  0.02  0.34  0.02  0.01 | 0.006  0.78  0.01  0.60  0.28 | 3.06  1.00  0.43  0.99  0.99 | 1.37-6.84  0.96-1.03  0.22-0.83  0.96-1.02  0.97-1.01 |
| Sleep disturbance   - Frailty - Age - Female - eGFR - Haemoglobin | 0.36  0.02  0.32  0.02  0.01 | 0.002  0.25  0.005  0.38  0.15 | 3.00  0.98  2.45  0.99  1.02 | 1.47-6.09  0.95-1.01  1.31-4.61  0.96-1.02  1.00-1.03 |
| Loss of appetite   - Frailty - Age - Female - eGFR - Haemoglobin | 0.83  0.03  0.53  0.02  0.01 | 0.31  0.10  0.25  0.06  0.31 | 2.33  1.06  1.85  0.96  0.99 | 0.46-11.85  0.99-1.13  0.65-5.26  0.91-1.00  0.96-1.01 |
| Tiredness   - Frailty - Age - Female - eGFR - Haemoglobin | 0.36  0.02  0.32  0.02  0.01 | <0.001  0.02  0.03  0.12  1.00 | 3.97  0.96  2.02  0.98  1.00 | 1.97-8.00  0.93-1.00  1.09-3.74  0.95-1.01  0.98-1.02 |
| Bone/joint pain   - Frailty - Age - Female - eGFR - Haemoglobin | 0.34  0.02  0.32  0.02  0.01 | <0.001  0.68  0.14  0.23  0.51 | 3.96  1.01  1.60  0.98  0.99 | 2.02-7.74  0.98-1.04  0.86-2.99  0.95-1.01  0.98-1.01 |
| Poor concentration   - Frailty - Age - Female - eGFR - Haemoglobin | 0.59  0.02  0.42  0.02  0.01 | 0.004  0.44  0.12  0.20  0.08 | 5.64  0.98  1.95  0.98  1.03 | 1.76-18.09  0.94-1.03  0.85-4.45  0.94-1.01  1.00-1.05 |
| Loss of muscle strength   - Frailty - Age - Female - eGFR - Haemoglobin | 0.45  0.02  0.34  0.02  0.01 | <0.001  0.25  0.61  0.01  0.24 | 5.87  1.02  1.19  0.96  1.01 | 2.45-14.06  0.99-1.06  0.61-2.32  0.93-0.99  0.99-1.03 |
| Breathlessness   - Frailty - Age - Female - eGFR - Haemoglobin | 0.49  0.02  0.34  0.02  0.01 | <0.001  0.60  0.77  0.68  0.07 | 6.09  1.01  1.10  0.99  0.98 | 2.33-15.94  0.97-1.05  0.57-2.14  0.96-1.02  0.97-1.00 |

**Supplementary Table 5. *Continued.***

|  | SE | Unadjusted  P Value | | Odds Ratio | | 95% CI | |
| --- | --- | --- | --- | --- | --- | --- | --- |
| Cramp/muscle stiffness   - Frailty - Age - Female - eGFR - Haemoglobin | 0.42  0.02  0.34  0.02  0.01 | 0.001  0.96  0.11  0.14  0.08 | 4.02  1.00  1.71  0.98  1.02 | | 1.77-9.14  0.97-1.03  0.89-3.30  0.95-1.01  1.00-1.04 | |  |
| *Restless legs*   - *Model P Value 0.63* | - | - | | - | | - | |
| Feeling cold   - Frailty - Age - Female - eGFR - Haemoglobin | 0.42  0.02  0.32  0.02  0.01 | <0.001  0.44  0.40  0.10  0.94 | | 4.60  0.99  1.31  0.98  1.00 | | 2.03-10.43  0.96-1.02  0.70-2.47  0.95-1.01  0.98-1.02 | |
| *Urinary frequency*   - *Model P Value 0.20* | - | - | | - | | - | |
